# Projected change in global fisheries revenues under climate change

**DOI:** 10.1038/srep32607

**Published:** 2016-09-07

**Authors:** Vicky W. Y. Lam, William W. L. Cheung, Gabriel Reygondeau, U. Rashid Sumaila

**Affiliations:** 1 Nippon Foundation-Nereus Program and Sea Around Us, Global Fisheries Cluster, Institute for the Oceans and Fisheries, The University of British Columbia, Vancouver, B.C., V6T 1Z4, Canada; 2 Fisheries Economics Research Unit and OceanCanada partnership, Global Fisheries Cluster, Institute for the Oceans and Fisheries and Liu Institute for Global Issues, The University of British Columbia, Vancouver, B.C., V6T 1Z4, Canada; 3 Nippon Foundation-Nereus Program and Changing Oceans Research Unit, Global Fisheries Cluster, Institute for the Oceans and Fisheries, The University of British Columbia, Vancouver, B.C., V6T 1Z4, Canada

## Abstract

Previous studies highlight the winners and losers in fisheries under climate change based on shifts in biomass, species composition and potential catches. Understanding how climate change is likely to alter the fisheries revenues of maritime countries is a crucial next step towards the development of effective socio-economic policy and food sustainability strategies to mitigate and adapt to climate change. Particularly, fish prices and cross-oceans connections through distant water fishing operations may largely modify the projected climate change impacts on fisheries revenues. However, these factors have not formally been considered in global studies. Here, using climate-living marine resources simulation models, we show that global fisheries revenues could drop by 35% more than the projected decrease in catches by the 2050 s under high CO_2_ emission scenarios. Regionally, the projected increases in fish catch in high latitudes may not translate into increases in revenues because of the increasing dominance of low value fish, and the decrease in catches by these countries’ vessels operating in more severely impacted distant waters. Also, we find that developing countries with high fisheries dependency are negatively impacted. Our results suggest the need to conduct full-fledged economic analyses of the potential economic effects of climate change on global marine fisheries.

Global marine fisheries landings are estimated officially at between 80 and 85 million t a year since 1990, with corresponding mean annual gross revenues fluctuating around USD 100 billion annually^1^. Accounting for unreported catches, a recent study estimated the likely “true” annual global catch to be about 130 million t^2^. The global fisheries sector supports the livelihoods of between 660 to 820 million people, directly or indirectly, which is about 10–12% of the world’s population^3^, if the dependents of fishers are taken into account. Globally, fish also provides more than 2.9 billion people with 20 percent of their animal protein needs^3^ and is a crucial source of micronutrients^4^. However, along with other non-climatic drivers such as changes in markets, demographics and overexploitation, climate change is considered to be a major challenge that will significantly shape the future of global fisheries. Several studies suggest that these non-climatic stresses and changes in management regimes may have a greater impact on fisheries than climate change in the short term^5^, while increasing uncertainty in climate poses a major threat to world fisheries in the long run^6^.

Changes in ocean conditions, including temperature, sea ice extent, salinity, pH, oxygen levels and circulation, lead to shifts in the distribution range of marine species^7^,^8^,^9^,^10^, changes in primary and secondary productivity, and shifts in timing of biological events^6^. Warmer temperatures may also lead to decreases in maximum body sizes of marine fishes^11^. The combined effects of the predicted distributional shift and changes in ocean productivity under climate change are expected to lead to changes in species composition^12^ and hence global redistribution of maximum catch potential (MCP), with projected increases in MCP in high latitudinal regions and decreases in the tropics^13^. These changes have large implications for people who depend on fish for food and income, and thus the contribution of fisheries to the global economy^14^,^15^.

The changes described above under climate change are bound to affect the economics of fishing through changes in revenues (price x landings), costs (fixed + variable costs)^16^ and fisheries subsidies^17^. In this study, we will, as a first step in understanding the potential economic impact of climate change, focus on modeling the effects of climate change on revenues through changes in the amount and composition of catches. Price dynamics are affected by the interplay between the supply and demand of seafood products. The preference of consumers and the development of other food supply sectors such as aquaculture may also affect the future price of seafood and therefore have the potential to alter the economic impact under climate change. Here, price dynamics are incorporated as exogenous factors and the effects on revenues are explored by conducting different scenario analysis on prices. These scenarios describe how future development of other production sectors in the economy would likely affect seafood prices.

Specifically, changes in total potential catches may not directly equate to changes in revenues from fisheries. Firstly, climate change may affect catches of species that command different prices in the market. Secondly, even though potential catches are expected to increase in some countries’ exclusive economic zones (EEZs), the fishing sector of these countries may still suffer if they include a substantial Distant Water Fishing fleet (DWF) that operates in foreign waters that are impacted by climate change.

Here, we examine the impacts of climate change on global fisheries revenues by combining the outputs of coupled atmospheric-ocean physical and biogeochemical Earth System Models (ESM) with Dynamic Bioclimate Envelope Models (DBEM)^9^. To explore the effects of different ESMs and DBEMs on the results, we use outputs from three ESMs that are available for the Coupled Models Intercomparison Project Phase 5 (CMIP5): the Geophysical Fluid Dynamics Laboratory Earth System Model 2 M (GFDLESM2M,) the Institute Pierre Simon Laplace (IPSL) (IPSL-CM5-MR) and Max Planck Institute for Meteorology Earth System Model (MPI-ESM MR) (Method).

The DBEM is a mechanistic species distribution model that links prediction of habitat suitability to their spatial and temporal population dynamics and eco-physiology^18^,^19^ (Method). We applied the DBEM to project changes in distributions, abundances and catches by the mid-21^st^ century for each of the 887 marine fish and invertebrate species on a half degree x half degree grid of the world ocean (280 EEZs and the high seas) under the Representative Concentration Pathways (RCPs) 2.6 and 8.5 (Method). RCP 2.6 and 8.5 represent the low emission “strong mitigation” and high emission “business-as-usual” scenarios, respectively. The sample of species represents 60% of total global average annual reconstructed catch in the 2000 s^2^. The outputs from DBEM were linked to the global marine catch and fisheries economic databases^1^,^2^,^20^ (Method) to project the impact on the revenues of each coastal country. This includes catches from fishing in EEZs and the high seas (Supplementary Information).

## Results and Discussion

**Figure 1:**
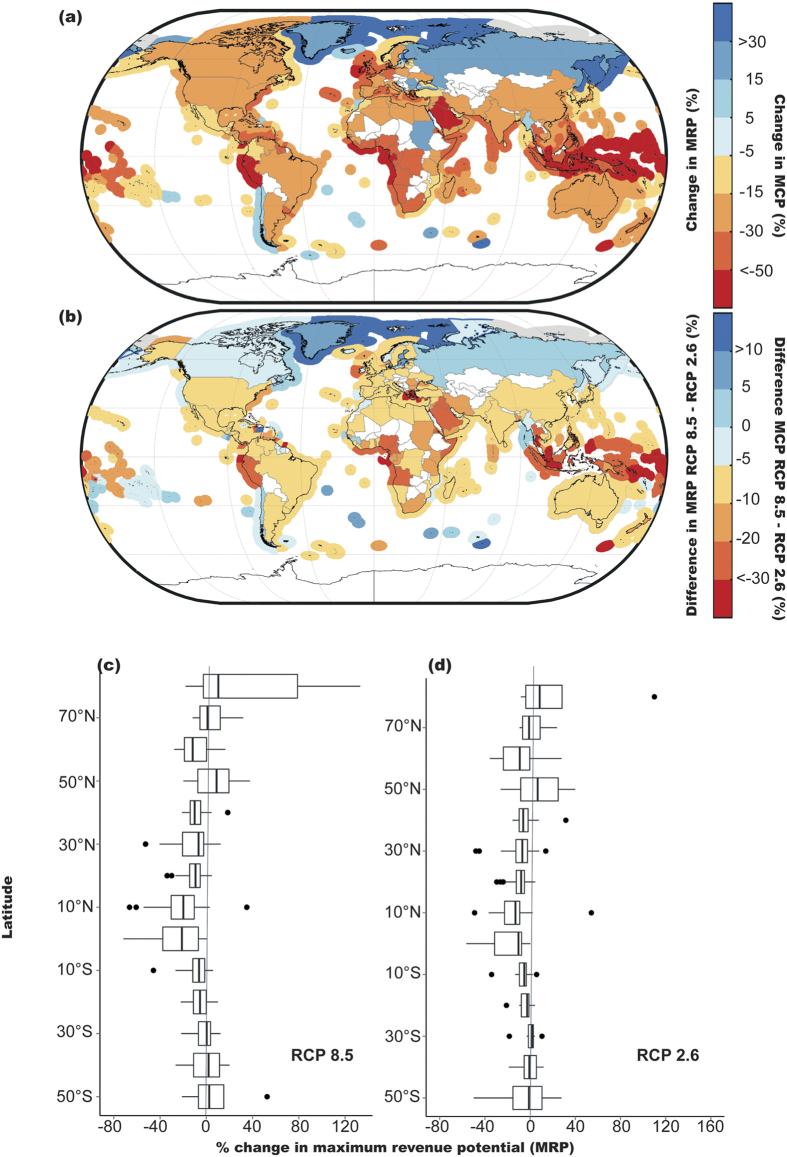
Impacts of climate change on MCP and MRP by the 2050 s (average between 2041–2060) relative to the 2000 s (average between 1991–2010): (**a**) mean percentage change in projected maximum catch potential (MCP) of 280 Exclusive Economic Zones (EEZs) and mean percentage change in projected MRP of 192 fishing nations in the 2050 s relative to the level in the 2000 s under RCP 8.5 scenario; (**b**) differences in percentage change in MCP and MRP between RCP 8.5 and RCP 2.6 scenarios in the 2050 s; (**c**,**d**) latitudinal zonal average of mean percentage change in fisheries MRP in different fishing countries under RCP 8.5 (**c**) and RCP 2.6 (**d**).

Assuming constant price, global MCP is projected to decrease globally by 7.7% (±4.4%, average across results from three ESMs) by 2050 (average between 2041–2060) relative to 2000 (average between 1991–2010) under the business-as-usual scenario (RCP 8.5). In contrast, global fisheries revenue (or landed value at Maximum Revenue Potential–MRP) is projected to decrease by 10.4% (±4.2%), i.e., about 35% more than the impact on MCP (Table 1) at a global scale. With an estimated total MRP of $100 billion, the variation in projected change in MRP between different ESMs ranges from US $ 6 to 15 billion, which seems small in one particular sector but may amplify when the economic impact of fisheries-dependent (direct and indirect) sectors are considered^21^. The percentage changes in MCP and MRP in the high seas are estimated at −3.2% and −4.8%, respectively, under RCP 8.5 while changes in EEZs are −8.2% and −11.4%, respectively. Under the strong mitigation scenario (RCP 2.6), the impact of climate change is lower than that under RCP 8.5, with MCP and MRP decreasing by an average of 4.1% (±3.8%) and 7.1% (±3.5%), respectively, by 2050 relative to 2000. Thus, the negative impact on MRP under RCP 8.5 is about 45.8% more relative to that of RCP 2.6 in the 2050 s. Our models also project percentage change in MCP of each of the 280 EEZs and MRP of each of 192 fishing nations in the 2050 s under RCP 8.5 (Fig. 1).

We explored the effects of different seafood price scenarios on future fisheries MRP. These price scenarios are based on the results from the United Nations’ “Fish to 2020” study^22^ that includes: (1) baseline; (2) faster aquaculture expansion; (3) slower aquaculture expansion; (4) lower China production; and (5) fishmeal and fish oil efficiency scenarios (Method and Supplementary Information). We compare projected MRP from these alternative price scenarios to the “constant price” scenario in which price stays the same as that in the 2000 s. The “constant price” scenario is different from the “baseline” price scenario as price under the latter changes from 8.7% to 34.8% in the seafood commodity groups (Supplementary Information).

Our projected MRP are most sensitive (i.e., with the largest changes from the “constant price” scenario) to the “slower aquaculture expansion” scenario (Supplementary Information). The increase in prices of all seafood commodity groups leads to an increase in MRP of approximately 7 and 9 times the magnitude of change under the “constant price” scenario for RCP 8.5 and RCP 2.6 scenarios, respectively (Table 2). Since global seafood production from aquaculture has been increasing at an annual rate of 8% in the past decade^23^, this scenario is unlikely to happen based on the current trend^23^. Only two of six price scenarios show negative impact on MRP (Table 2). Under the “faster aquaculture expansion” scenario, seafood prices of two seafood commodity groups (i.e., low value food fish and mollusks) decrease, resulting in a more substantial decrease (more than double) in fisheries MRP relative to the “constant prices” scenario (Table 2). The majority of price scenarios assume an increase in price by 7 to 57% by 2050 relative to 2000 that compensate for the projected decrease in catch and result in a projected increase in fisheries MRP even under the high emission scenario (11–40%).

The degree of economic impact also depends on how people value the future, which is represented by the discount rate^24^. With high discount rates, future values are given lower weight than current values while lower discount rates do the opposite. In this analysis, we use a zero discount rate that implicitly assumes a strong intergenerational consideration that weigh current and future values equally. For example, the difference in annual MRP between RCP 2.6 and 8.5 by 2050 is US$ 3.26 billion under a zero discount rate. However, with a 3% discount rate, the difference in annual MRP becomes US$ 0.74 billion only. The 3% and 0% discount rates can be viewed as the high and low bounds on discount rates, respectively, to be employed on long-run programs with intergenerational equity considerations^25^. Since our analysis focuses mainly on identifying spatial variations in MRP and vulnerability of fisheries under climate change, we employ 0% discount rate. Future studies that assess the economic impacts of climate change should select the appropriate discount rate according to the goal of the analysis to be carried out^26^.

Some countries are projected to see a large increase in MCP in their own EEZs but the percentage increase in their projected MRP is relatively low (Fig. 1). For instance, Greenland EEZ is expected to see a 58% increase in total MCP by the 2050 s, however, the expected increase in MRP is only 19% under the RCP 8.5 scenario. This is due to the decline in MCP of some highly economically valuable species, such as Atlantic salmon (*Salmo salar*) (−70%) and European plaice (*Pleuronectes platessa*) (−64%). Thus, total MRP from fisheries are not only dependent on the amount of catch, but also on catch composition and associated prices. Also, Greenland fisheries do not only fish in its own EEZ, but also along Canada’s east coast. As a result, the projected positive impact of the MCP in a country’s EEZ may not necessarily mean a positive impact on its economy, and *vice versa*. For the top 10 countries with the highest catch volume from Distant Water Fishing (DWF), their overall MCP outside their own EEZs is projected to decrease by 1.5% (±2.4%) from the current status quo even though their domestic MCP is projected to increase by 4.8% (±0.4%) under the RCP 8.5 scenario. In terms of MRP, the negative impact generated by a country’s DWF is 5.2% (±2.2%), whereas the MRP generated by the country’s domestic catch stays virtually unchanged (0.5 ± 3.1%). Therefore, countries with DWF are not only affected by the change in MCP in their own EEZ, but also affected by the climate change impacts in other EEZs and the high seas.

**Figure 2:**
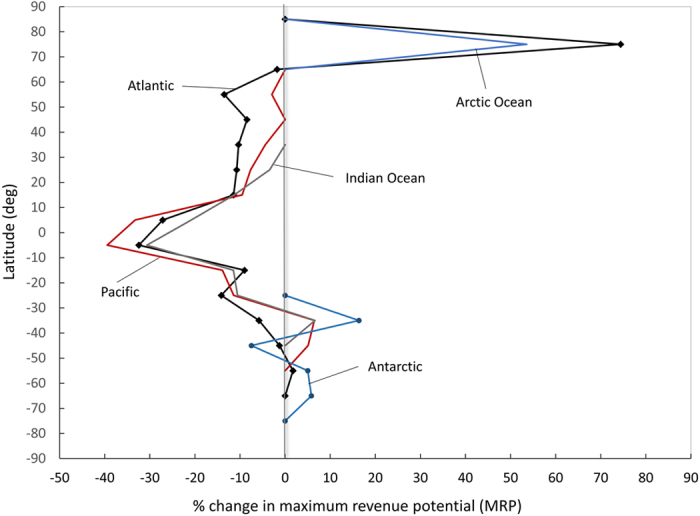
Percentage change in fisheries maximum revenue potential (MRP) in different ocean basins at different latitudes. Blue line represents Arctic Ocean; blue dotted line represents Antarctic Ocean; black dotted line represents Atlantic Ocean; grey line represents Indian Ocean; and red line represents Pacific Ocean.

The impacts on MRP vary for EEZs in different latitudes and ocean basins (Fig. 1 and Fig. 2). Impacts in the tropics (10°N to 10°S) are projected to be the highest (Fig. 1), with MCP and MRP decreasing by 38% and 33%, respectively, under RCP 8.5. Overall, 80% of EEZs worldwide (224 EEZs) are projected to show a decline in MCP. The greatest positive impact is projected to be in the Arctic Ocean (regions higher than 75°N) and the Northern Atlantic (regions higher than 70°N) where MRP are projected to increase by 71% and greater than 100%, respectively (Fig. 1). This latitudinal gradient is consistent with the findings from previous studies^13^,^15^,^21^. The greatest negative impact is in tropical regions with EEZs in the central Pacific and central Atlantic being affected the most (Fig. 2). The EEZs with the largest average decrease in MCP mostly belong to small island countries, for example, Tuvalu (−79%) and Kiribati (−70%). These countries are highly dependent on fisheries for food and livelihoods and their marine ecosystems are more vulnerable to climate change^4^,^27^. In contrast, EEZs in higher latitude regions are projected to see positive impacts. In the Arctic, the melting of sea ice may create new fishing opportunities for non-Arctic fishing countries but may pose a threat to the Arctic countries’ fleets, which are currently primarily artisanal and subsistence-based.

**Figure 3:**
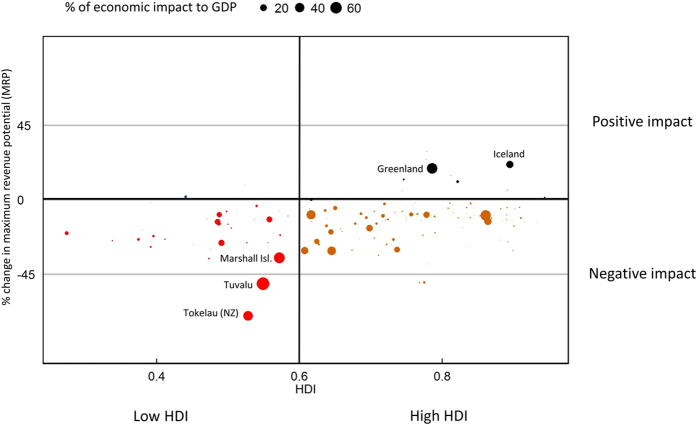
Percentage change in fisheries Maximum Revenue Potential (MRP) is mapped against Human Development Index (HDI) of countries. The bigger the size of the bubble the larger the percentage of economic impact of the fisheries sector to the total Gross Domestic Product (GDP).

Climate change is projected to have negative impacts on the MRP of 89% of the world’s fishing countries (170 countries) (Fig. 3). Most of the low Human Development Index (HDI) countries are coastal low-income food deficit countries (LIFDCs) – almost all of them are projected to see decreases in the MRP under RCP 8.5. Coastal LIFDCs are heavily dependent on fish catches to meet their animal protein and nutritional needs. Despite the relatively low level of per capita fish consumption in these countries (10 kg/year), the contribution of fish to animal protein intake is relatively large (24%)^3^. LIFDCs also rely on fish and fisheries as a source of income and job opportunities. The value-added from fisheries allows people to purchase high calorie staples such as rice and wheat, and other nutritious food such as vegetables and meat. Negative impacts on the catch and total MRP obtained by these countries may have greater implications on food security than the impact on high HDI countries^28^. These impact is even more important in countries (e.g., in Africa; Southeast Asia) where other natural resource sectors such as agriculture are projected to see large declines in yield under climate change^29^. Furthermore, in many low HDI countries, the marine resources within their own EEZs are exploited and threatened by distant water fishing (DWF) fleets from other fishing countries^30^, for instance, countries in West Africa. Although the change in catch under climate change may also have impacts on these foreign fishing countries, these countries can either stop fishing in these EEZs or shift to other fishing grounds. However, small-scale fisheries in developing countries are those who would suffer from the impacts identified the most as they have relatively lower capacity to adapt to the change^27^.

The impact on revenues may have large implications for the whole economy through indirect economic effects of fisheries on activities such as boat building/maintenance, equipment supply and the restaurant sector. In some countries, fisheries constitute a base industry to the whole national economy. The current secondary and induced economic impacts of fisheries sector in each country are estimated by applying the national fishing output multipliers^31^ (Supplementary, Table S7) to the current actual fisheries revenues. An output multiplier is a coefficient that is multiplied by the output of an economic activity to obtain the total contribution to the economy. The fisheries sector is important to a country if its contribution to the national Gross Domestic Product (GDP) of the country is high. This is because the negative impacts of climate change on these countries would be more significant than in countries where fisheries activities makes only a minor contribution to the economy. So, low HDI countries that heavily depend on fisheries for their national income are mostly more vulnerable under climate change (Fig. 3). Examples of such vulnerable countries are Tokelau, Tuvalu and Marshall Islands (red dots in Fig. 3). In contrast, fisheries in many of the countries that would benefit under climate change, mostly high HDI countries, have small contribution to national economies.

Overall, the qualitative conclusions of this study are robust to structural uncertainties of the DBEM (Table S3). Across different algorithms of predicting species’ habitat suitability (DBEM-Basic, DBEM-Aqua and DBEM-Maxent)^32^, the global projected changes in MCP and MRP under RCP 8.5 range from −3.6% to −8.2% and −4.1% to −9.6%, respectively. Their coefficient of variation (COV) for MCP and MRP, calculated from the standard deviation to mean ratio, are 45% and 40%, respectively. Variability of projections from different DBEMs are slightly lower than from different ESMs, the latter have coefficient of variations of 57% and 41% for MCP and MRP, respectively. Future studies, such as those planned by the Fisheries and Marine Ecosystem Model Inter-comparison Project (FishMIP), should more thoroughly explore the variability of projections from different models^33^.

The global scope of this study requires us to make various assumptions in representing the dynamics of fisheries revenues and biophysical systems – these assumptions can incrementally be relaxed in future studies. Fishing revenues can capture the impact on both the fishing industry and the consumer but only partially because fishing costs are not included. To fully examine the economic impacts of climate change on society through fisheries, future studies should include analysis of both consumer and producer surpluses. Actual catch might not necessarily equal the MCP because catch may be influenced by other factors such as fisheries management, fishing cost and subsides. Since this study focuses on the effect of climate change on catch and revenue potential, we keep the fishing effort constant (at a level to achieve Maximum Sustainable Yield), thus, catch is solely driven by biology.

Until now, there has been only little attempt at regional scale modelling of fishing effort dynamics. Future studies should also consider the influence of different policies such as the quota constraints imposed by the major tuna regional fisheries management organizations (RFMOs) and adaptive responses of the fishing fleets. In our study, DWF countries are projected to suffer higher adverse impacts on their MRP under climate change and constant fishing effort scenario. Although DWF fleets may be capable of switching to other less impacted EEZs, the cost for this shift (e.g., contract negotiations with new countries) and geopolitical limitation may constrain their ability to do so. Other human and socio-economic responses to climate change may also affect future economic impacts. For example, adaptation responses of the fisheries such as the development of new fisheries that target invading species may modify the realization of potential catches in the future. Thus, changes in fleet size and fishing effort dynamics, and the variability in ex-vessel prices of marine species, under changing oceans need to be explored.

## Conclusion

Our study highlights the impacts of climate change on society through its impact on revenues from fishing as a result of the interplay between ecology and fishing patterns. Our results suggest that the negative impact on MRP under the “faster aquaculture expansion” scenario is higher than the change under the “constant price” scenario. This suggests that we have to carefully consider development of aquaculture as a way to adapt to climate change impacts on marine capture fisheries. The results also indicate that the countries that are most highly exposed to fisheries revenue impacts due to climate change have lower adaptive capacity to absorb these changes. We find that the projected impacts on revenues are relatively robust to climate and structural uncertainty, but not to the range of discount rates and prices explored in this contribution. Future work is needed to assess the full economic effects of mitigating or not mitigating GHG emissions.

## Methods

Distributional shifts in exploited marine species were investigated using a Dynamic Bioclimate Envelope Model (DBEM)^9^. Change in catch was estimated based on differences in the MCP by the 2050 s projections of catch using an empirical model^13^. Then, we combined projected catches with economic parameters including ex-vessel fish prices^1^,^20^ to compute the percentage change in revenues in each country under climate change. Details of DBEM, empirical and economic models are provided below and in the Supplementary Material.

### Climate scenarios

We used the RCP 2.6 and 8.5 scenarios, which represent low-range and high-range of GHG emissions, respectively. Projected changes in ocean conditions (including sea surface temperature, sea bottom temperature, salinity, sea surface advection, sea ice extent, oxygen concentration and net primary productivity) were based on outputs from three different Earth System Models (ESM). Multimodel ensemble is used to mitigate model uncertainty. These ESMs include Geophysical Fluid Dynamics Laboratory Earth System Model 2 M (GFDL ESM2M), IPSL-CM5-MR and MPI-ESM for the RCP 8.5 scenario. For RCP 2.6 scenario, the projected changes were based on outputs from the GFDL ESM2M as the outputs from the model are in the middle of the range projected by the different ESM models applied. The outputs were re-gridded onto a 0.5° x 0.5° grid map of the world ocean using the nearest neighbour method, which interpolated the missing values by selecting the value of the nearest point, while missing values in some grid cells were interpolated using bilinear interpolation.

### Biological model

#### Projecting future species distribution and maximum catch potential (MCP) under climate change

Distributions of 693 demersal and 194 pelagic marine fish and invertebrate species in the recent few decades on a 0.5° latitude × 0.5° longitude grid of the world’s ocean were determined using an algorithm that was based on the species’ depth range, latitudinal range, habitat preferences and broadly known regions where the species occurs. The parameter values of each species were obtained from online databases such as FishBase (www.fishbase.org) and SealifeBase (www.sealifebase.org).

Based on the current distribution, the DBEM simulates changes in distribution of abundance and MCP of fishes and invertebrates over time and space driven by projected changes in ocean conditions, with consideration of physiological and ecological effects of changes in ocean properties and density-dependent population growth and movement. The details of DBEM are described in Cheung *et al*.^9^ and in the Supplementary Material. The carrying capacity of each species in each grid cell varies positively with its habitat suitability, which is predicted by sea surface temperature, salinity, oxygen content, sea ice extent (for polar species) and bathymetry. The model simulated changes in relative abundance of a species in each spatial cell at each time step by incorporating the intrinsic population growth, and settled larvae and net migration of adults from surrounding cells using an advection-diffusion-reaction equation. The model also simulates how changes in temperature and oxygen content would affect growth of the individuals.

Based on the projections of the future distribution of the selected marine species and the projected primary production from the outputs of the ESMs, we estimated the annual MCP using the published empirical model of Cheung *et al*.^13^. MCP is a proxy of the maximum sustainable yield (MSY), calculated from MCP = F_MSY_ × B where F_MSY_ is the fishing mortality required to achieve MSY (approximated from F_MSY_ = natural mortality rate of the stock) while B is the projected biomass in each year and spatial cell. In each grid cell, the projected catch of the species was allocated to each fishing country based on cell-based data from the reconstructed catch database of the *Sea Around Us* (www.searoundas.org), which has the information of current catch of each species in each cell by each fishing country. Then, total MCP of EEZ was calculated from the sum of catch in cells belonging to that EEZ. The projected percentage changes in MCP in each EEZ in the 2050 s relative to current status (2000 s) was calculated using the total MCP of all species caught in a given EEZ.

#### Exploring model and structural uncertainties

We used a multi-model ensemble to explore sensitivity of the assessment and address the uncertainties of different ESMs. The final projected changes in MCP are the average outputs of all the three ESMs (i.e., GFDL-Basic, IPSL-Basic and MIP-Basic). For assessing the uncertainty of the biological models, we applied alternative versions of DBEM. Other than using the spatial distribution model (SDM) developed by the *Sea Around Us*, we used Maxent^34^ and AquaMaps^32^ to address the sensitivity of relative MCP and latitudinal range shifts obtained under RCP 8.5 to alternative spatial distribution model approaches and their underlying assumptions. The results from the models using different SDMs including DBEM-Basic, DBEM-Maxent and DBEM-AquaMaps are shown in the Supplementary Material (Table S3). The standard deviation of the percentage of change in MCP and fisheries MRP are 2.4 and 2.8%, respectively, and indicates that the uncertainty from different SDMs is smaller than the uncertainty among different ESMs.

#### Estimating revenue parameters

Projected revenue is the product of species-country ex-vessel price and projected MCP of each species. This study assumes that the real ex-vessel price (i.e., after adjusting for inflation) to be constant throughout the study period because the projection of future price is limited by data availability and model complexity. It is worth noting that the real ex-vessel fish prices have remained relatively stable since 1970^1^ even though they are likely to increase in the future^22^. To test the effect of this constant real price assumption, we carried out sensitivity analysis to determine how changes in price are likely to affect the results of our study.

#### Exploring revenue uncertainties

For testing revenue uncertainties, we projected the price trends of different groups of marine species based on the available price information under different projected price scenarios^22^ (Supplementary, Table S4). In each production projection scenario, we estimated the future price of each species based on the forecasted price of each commodity group (Supplementary, Table S5). For example, the price of low value food fish decreases by a total of 26% in the “faster aquaculture expansion” scenario in 2050 over 2000 level. Then, the future fisheries revenues were estimated using the projected price and the sensitivity of the results to this assumption is evaluated.

## Additional Information

**How to cite this article**: Lam, V. W. Y. *et al*. Projected change in global fisheries revenues under climate change. *Sci. Rep.* **6**, 32607; doi: 10.1038/srep32607 (2016).

## Supplementary Material

Supplementary Information

## Figures and Tables

**Table 1:** Projected percentage change in global maximum catch potential (MCP) and fisheries maximum revenue potential (MRP) in the 2050 s from the current status under different climate change scenarios.

	Model uncertainty				
	% change in maximum catch potential				
	GFDL	IPSL	MIP	**Mean**	Standard deviation
RCP 2.6	−1.66	−8.49	−2.03	**−4.06**	3.84
RCP 8.5	−4.44	−12.66	−6.02	**−7.71**	4.36
	% change in fisheries maximum revenue potential				
RCP 2.6	−5.07	−11.15	−5.12	**−7.11**	3.50
RCP 8.5	−6.88	−15.03	−9.21	**−10.37**	4.20

**Table 2:** Percentage change in fisheries maximum revenue potential (MRP) in the 2050 s relative to the 2000 s under different price scenarios and projections.

	% change in fisheries MRP in the 2050 s relative to the 2000 s							
	Constant price	Baseline	Faster aquaculture expansion	Lower China production	Fish mean and fish oil efficiency	Slower aquaculture expansion	Mean	Standard deviation
RCP 8.5	−6.88	12.78	−14.77	13.77	10.75	39.58	9.20	18.93
RCP 2.6	−5.07	14.88	−13.34	15.88	12.81	42.36	11.25	19.37
