# Supplementary material for: Projected change in global fisheries revenues under climate change
Source: Sci Rep. 2016 Sep 7;6:32607. doi: 10.1038/srep32607 (PMC5013467; doi:10.1038/srep32607)
Supplement: Supplementary Information [file srep32607-s1.pdf]

Supplementary Information

Manuscript title: Projected change in global fisheries revenues under climate change

Authors: Vicky W. Y. Lam, William W. L. Cheung, Gabriel Reygondeau and U. Rashid Sumaila

Climate scenarios and models

To explore future changes in potential fisheries catches during the period from 1971 to 2060, environmental outputs of several atmospheric-ocean physical models coupled with biogeochemical models have been acquired. Annual average values of surface and bottom sea water temperature (°C), oxygen concentration ($ml.L^{-1}$), salinity, net primary production ($mgC.km^2.year^{-1}$), surface advection (zonal and meridional vectors in $m.sec^{-1}$), and percentage of sea ice coverage (%) were gathered from the outputs of the Geophysical Fluid Dynamics Laboratory Earth System Model (GFDL ESM2M)[1], the Institut Pierre Simon Laplace model[2] (IPSL-CM5-MR) and from the Max Planck Institute for Meteorology model (MPI-ESM)[3]. Each environmental output is re-gridded onto a regular grid of 0.5°using the nearest neighbour method and values in some coastal cells were extrapolated using bilinear extrapolation.

The different types of uncertainties associated with the use of climate models have been explored in this study. Firstly, to evaluate the uncertainties associated to future climate projections due to potential changes in Green House Gas (GHG) emissions, two representative concentration pathways scenario (RCP 2.6 and 8.5) are gathered for each environmental variables from the

GFDL-ESM2M. The first scenario (RCP2.6) emphasizes the radiative forcing trajectory peaked at $3W/m^2$ before 2100 and then followed by a decline to $2.6W/m^2$ by 2100. The second scenario (RCP8.5) is a high emission scenario with rising radiative forcing pathway leading to $8.5W/m^2$ by 2100[4]. GFDL ESM2M model has been selected as the main model because of its good skills in representing climate changes relating to surface circulation particularly in simulating surface temperature, salinity, and height patterns, tropical Pacific circulation and variability; and Southern Ocean dynamics[1]. Secondly, to explore the inter-model uncertainties, our projections were also driven by the two other IPCC-class Earth System Models (IPSL-CM5-MR and MPI-ESM-MR).

Biological Model

The Dynamic Bioclimate Envelope Model (DBEM) simulates changes in the distributions and relative abundances of the marine species under the climate change scenarios.

Current species distribution

Current species' distributions in the recent decade (1971 – 2000) were obtained using a spatial distribution model developed by the *Sea Around Us*[5,6](www.searoundus.org). The model determines distributions of marine fishes and invertebrates by predicting the relative abundance of a species on a 30' latitude x 30' longitude grid based on the species' (i) known FAO area(s); (ii) latitudinal range; (iii) polygons encompassing their known occurrence regions; (iv) depth range; and (v) habitat preferences (for detailed methodology, please refer to Close *et al.*[5]).

Projecting future distribution

DBEM simulates how changes in temperature and oxygen content (represented by O_2 concentration) would affect growth of marine fishes and invertebrates. This model was done for each species. The model algorithm is derived from a growth function:

$$\frac{dB}{dt} = H \cdot W^a - k \cdot W \qquad (1)$$

where *H* and *k* are coefficients for anabolism and catabolism, respectively. *B* is the biomass of each species. Anabolism scales with body weight (*W*) with an exponent $a < 1$, while catabolism scales linearly with *W*. Solving for dB/dt = 0, we obtained $H = k$ where W_∞ is the asymptotic weight.

Equation (1) can be integrated to a generalized von Bertalanffy Growth Function (VBGF; Pauly 1981):

$$W_t = W_\infty \cdot [1 - e^{-K \cdot (t - t_0)}]^{\frac{1}{1-a}} \qquad (2)$$

where *K* is the von Bertalanffy growth parameter.

For simplification, we assume that $a = 0.7$, although empirical studies show that *a* generally varies from 0.50 to 0.95 between fish species[7,8] , with 2/3 corresponding to the special or standard VBGF.

Moreover, metabolism is temperature dependent and aerobic scope is dependent on oxygen availability in water and maintenance metabolism is affected by physiological stress (e.g., increased acidity). Thus:

$$H = g \cdot [O_2] \cdot e^{-j_1/T} \qquad (3a)$$

$$k = h \cdot [H^{+}] \cdot e^{-j_2/T} \cdot \qquad (3b)$$

where $j = E_a/R$ with E_a and R are the activation energy and Boltzmann constant, respectively, while T is temperature in Kelvin. In addition, the aerobic scope of marine fishes and invertebrates decreases as temperature approaches their upper and lower temperature limits[9]. The coefficients g and h were derived from the average W_∞, K and environmental temperature (T_o) of the species reported in literature:

$$g = \frac{{W_\infty}^{1-a} \cdot K}{[O_2] \cdot e^{-j_1/T_0}} \qquad (4a)$$

$$h = \frac{K/(1-a)}{[H^{+}] \cdot e^{-j_2/T_0}} \qquad (4a)$$

where $H = k$ and $k = K$ / (1-a) (eq. 1 and 2).

The model predicts changes in VGBF parameters according to changes in temperature, oxygen, and pH in the ocean relative to the initial conditions, as:

$$W_\infty = \left(\frac{H}{k}\right)^{\frac{1}{(1-a)}} \qquad (5a)$$

$$K = k \cdot (1 - a) \qquad (5b)$$

Based on the computed VGBF parameters and environmental conditions, the model determined change in carrying capacity in each 30' latitude x 30' longitude cell. The model identifies the 'environmental preference profiles' of the studied species, defined by the sea water temperature (bottom and surface), depth, salinity, distance from sea-ice and habitat types. Preference profiles are defined as the suitability of each of these environmental conditions to each species, with suitability calculated by overlaying environmental data (1970-2000) with distribution

range of relative abundance of the species[10]. Temperature is not used for predicting baseline current distribution[11]. For example, for each species, the model calculated a temperature preference profile for the adult and the pre-recruit phases based on the relative abundance and the computed recruitment strength of the species. Sea surface temperature is then used for temperature preference profiles for pre-recruit phase while bottom temperature is applied to preference profiles for adult demersal species. Moreover, the carrying capacity is expressed as a function of expected biomass per recruit and recruitment. Expected biomass per recruit was determined by a size-based population model. Thus, a change in species' carrying capacity in each spatial cell is dependent on its calculated theoretical relative abundance, environmental preferences and net primary production. Natural mortality rate (M) and length at maturity are determined from published empirical equations (see Cheung *et al.*[12] for details). Initial relative recruitment strength (R) is calculated from the initial relative abundance (A, normalized across the 30' x 30' degree resolution grid) and calculated biomass per recruit (BPR) in each cell, as

$$BPR = c \cdot A/R \quad (6a)$$

where c is a constant that scales from relative abundance to absolute abundance. Thus,

$$R = c \cdot A/BPR \quad (6b)$$

and

$$A = BPR \cdot R/c \quad (6c)$$

The model simulates changes in relative abundance of a species by:

$$\frac{dA_i}{dt} = \sum_{j=1}^{N}(L_{ji} + I_{ji}) + G_i \quad (7)$$

where A_i is the relative abundance of a 30' x 30' cell i, G is the intrinsic population growth and L_{ji} and I_{ji} are settled larvae and net migrated adults from surrounding cells (j), respectively.

Intrinsic growth is modeled by a logistic equation:

$$G_i = r \cdot A_i \cdot (1 - \frac{A_i}{A_{\infty,i}}) \quad (8)$$

where r is the intrinsic rate of population increase. The model explicitly represents larval dispersal through ocean current with an advection-diffusion-reaction model (see Cheung *et al.*[10] for details).

$$\frac{\partial Lav}{\partial t} = \frac{\partial}{\partial x}\left(D\frac{\partial Lav}{\partial x}\right) + \frac{\partial}{\partial y}\left(D\frac{\partial Lav}{\partial y}\right) - \frac{\partial}{\partial x}(u \cdot Lav) - \frac{\partial}{\partial y}(v \cdot N) - \lambda \cdot Lav \quad (9)$$

where change in relative larvae abundance over time ($\partial Lav/\partial t$) is determined by diffusion (i.e., the first two terms on the right-hand side of eq. 9) and current-driven movements (i.e., the third and fourth terms of eq. 9). Diffusion is characterized by a diffusion parameter D, while advection is characterized by the two current velocity parameters (u, v) which describe the east-west and north-south current movement.

Maximum Catch Potential

We applied a constant fishing mortality rate across the geographic range of each species. Specifically, to predict the theoretical maximum potential catches (MCP), we assumed that fishing

was approximately at maximum sustainable yield (MSY) throughout the simulation. Given that logistic population growth was assumed in all versions of DBEM, fishing mortality rate at MSY for each species was equal to half of the intrinsic population growth rate of the species. i.e., $F = r/2$. The resulted outputs were the MCP of each species in each grid cell at each time step. To allocate the MCP of each species to each fishing country in each cell, we used the current cell-based catch data from the reconstructed catch database developed by *Sea Around Us* (www.seaaroundus.org). In the *Sea Around Us* catch data, the historical and current catch data of each marine species was allocated to each half degree cell using three main components: 1. the catch data; 2. the fishing access observations/agreements, and 3. the biological taxon distributions[13]. MCP of each species in each cell is calculated by:

$$MCP_{ijk} = MCP_{ik} * \frac{C_{ijk}}{\sum_{j=1}^{m}(C_{ijk})} \qquad (10)$$

where MCP_{ijk} is the maximum catch potential of species i by country j in a cell k and MCP_{ik} is the total maximum catch potential of species i in a cell k. C_{ijk} is the current catch (average from year 1991 to 2010) of species i by country j in a cell k.

Table S1. Parameters of the DBEM.

Parameters	Symbols	Unit
Abundance	A	No. of individuals
Activation energy	E_a	
Area the geographic range of a species	A	
Asymptotic weight	W_∞	
Biomass	B	t
Biomass per recruit	BPR	t
Body weight	W	
Bolzmann constant	R	
Change in relative larvae abundance over time	$(\partial Lav/\partial \mathrm{t})$	
Coefficient for anabolism	H	
Coefficient for catabolism	k	
Coefficients derived from the average W_∞, K and environmental temperature (T_o) of the species	g, h	
Diffusion coefficient	D	$m \cdot s^{-1}$
East-west current velocity	u	$m \cdot s^{-1}$
Initial relative recruitment strength	R	
Intrinsic population growth	G	$year^{-1}$
Intrinsic rate of population increase	r	
Maximum sustainable yield	MSY	t
Natural mortality rate	M	$year^{-1}$
Net migrated juveniles and adults from surrounding cells j to cell i	I_{ij}	count
North-south current velocity	v	$m \cdot s^{-1}$
Number of grid cells	N	Count
Primary production within its exploitable range	P	

Proportion of exploitable range relative to the geographic range of a species	α	
Seawater temperature	T	Kelvin
Settled larvae	L	
Trophic level	λ	
von Bertalanffy growth parameter	K	

DBEM – alternative structure

We applied alternative versions of DBEM for assessing the structural uncertainty of the projections. Other than using the spatial distribution model (SDM) developed by *Sea Around Us*, we used two other species distribution models, Maxent and AquaMaps, that are generative statistical procedures to determine species' environmental envelopes from species occurrence data and a suite of environmental variables[14]. These two models were applied to predict the current distribution of marine species using 30-year averaged environmental data center on 1985 (1970 – 1999). Then, we applied the oceanographic variables from the GFDL ESM2M to project species distribution using the 2 spatial distribution model. For details of these two models, please refer to the supplementary information of Jones & Cheung[14] and Cheung *et al*. in press[15]. The models that we used to address the uncertainty of the ESMs and the structural uncertainty of the projections are summarized in Table S2. The results from the DBEM using different SDMs are shown in Table S3.

Table S2. Climate scenarios used in this study.

			Structural uncertainty of projections		Model uncertainty	
Climate scenarios	RCP 2.6	RCP 8.5				
Earth System Models (ESM)	GFDL	GFDL	GFDL	GFDL	IPSL	CMIP
Spatial distribution models	Sea Around Us	Sea Around Us	AquaMaps	Maxent	Sea Around Us	Sea Around Us

Table S3. Percentage change in the global maximum catch potential and maximum revenue potential in fisheries from the DBEM using different spatial distribution models under RCP 8.5 scenario in the 2050s from the current status (2000s).

	% change in global maximum revenue potential in fisheries in the 2050s from the current status (2000s)				
Earth System Model	GFDL			**Mean**	Standard deviation
Spatial distribution models	Basic	AquaMaps	Maxent		
Maximum catch potential	-4.4	-8.2	-3.6	-5.4	2.4
Maximum revenue potential	-6.9	-9.6	-4.1	-6.9	2.8

<u>Estimating economic parameters</u>

Fisheries revenue

Total global revenues is the product of ex-vessel price (P) and landing in the case of commercial fisheries. In our model, the global total maximum revenue potential (MRP) in fisheries can be expressed as:

$$MRP = \sum_{j=1}^{m}[\sum_{i=1}^{n}(P_{ij} * MCP_{ij})] \quad (11)$$

where P_{ij} is ex-vessel price and MCP_{ij} is the current maximum catch potential (the model MCP) of species *i* caught by country *j*. The total fisheries MRP of each country *j* was first computed by summing up MRP (i.e., modelled landed values) of *n* species caught from its EEZ *j*, other EEZs and the high seas. The current total MRP was estimated using a 20-year (from 1991 – 2010) average ex-vessel prices of each species in 2005 real dollars[16,17] by each country and the 20-year average MCP data from the DBEM. Then, the global total fisheries MRP was obtained by summing up the fisheries MRP of all countries, where the total number of fishing countries is *m* (*m* = 192).

Projected total MRP (*MRP'*) is the product of ex-vessel price (*P'*) and projected MCP (*MCP'*) of each species and can be expressed as:

$$MRP' = \sum_{j=1}^{m} [\sum_{i=1}^{n} (P'_{ij} * MCP'_{ij})] \qquad (12)$$

The ex-vessel price of each species *i* in each country *j* (P'_{ij}) was assumed to be constant through time, although fish prices could be influenced by local markets, the global supply of fish, preference of consumers, prices of alternative products on the market and also the abundance of targeted species[18-22]. The projected imbalance between fish supply and demand might also lead to increases in fish price[23,24]. This study assumes that the real ex-vessel price (after adjusting for inflation) to be constant throughout the study period because the projection of future price is limited by data availability and model complexity. Also, real ex-vessel fish prices have remained relatively stable since 1970[17,25]. Although real fish prices are likely to rise in the future, for example, fish prices were projected to increase by about 6 – 15% over the 1997 level by 2020[26], the constant price assumption allows us to understand the general pattern of the impact of climate change on

global fisheries economics before a more detailed price projection model is available[25]. Meanwhile, sensitivity analysis is carried out to explore how changes in prices are likely to affect the results derived from our study.

Estimating economic uncertainties

It is expected that change in price trends is crucial to the degree of impact of climate change on fisheries and its subsequent economics. Therefore, we tested the sensitivity of our results to price based on available price information. We adopted the project scenarios described in the IMPACT model[26] as our catch scenarios in this analysis. Table S4 describes the details of each scenario. Then, the price ranges from 1997 to 2020 provided in literature[26] were used to project the price range from 2000 to 2050 of each scenario in our analysis. We assumed that the rate of price change is constant throughout the time period. Then, the annual percentage change in price was calculated by dividing the projected total change in price by the total number of years from 1997 to 2020 (i.e., 24 years). By assuming the price would increase at the same rate from 2000 to 2050, we then estimated the total price change in 2050 relative to the level in 2000. The projected percentage of total price change of each commodity group in 2050 is shown in Table S5.

Determine the high price and low price species

Since the SAU catch database does not associate with the commodity groups, we allocated different marine species to the 4 commodity groups listed in Table S5 based on the functional groups that they belong to and also their price ranges. For finfish, we segregated them into low value and high value groups based on their current ex-vessel price. We first took the median price of all the exploited marine fish species (i.e., US$ 1,924) and then used that as the threshold for

segregating low and high values fish species. For any fish species with prices lower than this threshold price, we considered them as low price food fish and vice versa.

Table S4. Description of production scenarios.

	Scenario	Description
1.	Baseline	Judged to be the most plausible set of assumptions.
2.	Faster aquaculture expansion	Production growth trends, excluding supply response to price change, for all four aquaculture output aggregate commodities are increased by 50 percent relative to the baseline scenario.
3.	Lower China production	Chinese capture fisheries production is reduced by 4.6 mmt in base year 1996–98 following Watson and Pauly (2001). Consumption is reduced an identical amount to maintain balance. Reductions are spread proportionately among fish commodities. Income demand elasticities, production growth trends, and feed conversion ratios are adjusted downward, consistent with the view that actual growth in production and consumption over past two decades was in fact slower than reported.
4.	Fishmeal and oil efficiency	Feed conversion efficiency for fishmeal and fish oil improves at twice the rate specified in the baseline scenario.
5.	Slower aquaculture expansion	Production growth trends, excluding supply response to price change, for all aqua culture commodities is decreased by 50 percent relative to the baseline scenario.

Source: Delgado (2003)[26]

Table S5. Projected total change in prices under different production scenarios, 2000 – 2050.

	Projected total change in price (%) , 2000 – 2050				
Seafood groups	Base line	Faster Aquaculture expansion	Lower China production	Fishmeal and fish oil efficiency	Slower aquaculture expansion
Low value food fish	13.04	-26.09	13.04	10.87	54.00
High-value finfish	32.61	19.57	34.78	30.43	41.30
Crustacean	34.78	8.70	41.30	32.61	56.52
Mollusks	8.70	-34.78	6.52	6.52	54.00

Project landed values under different production scenario

With the projected total price change of each commodity group, we projected the price of each marine species in the 2050s under different production scenarios. The future fisheries MRP were then calculated with these projected prices and projected catch under the RCP 8.5 and RCP 2.6 scenarios. The percentage changes in the fisheries MRP under different production scenarios are shown in Table 2 in the main text.

Dependency of a country's national economy on its fisheries

We used the percentage of current total economic impact of fisheries sector to a country's national Gross Domestic Product (GDP) to determine the importance of the fisheries sector to a country's economy. To capture important contributions of fish populations to the whole economy, the value created through the production chain was also captured in this study. The added value or impact through the fish value chain is the indirect economic effects of fisheries due to their impact on activities such as boat building/maintenance, equipment supply and the restaurant sector[27]. In most of the countries, fisheries constitute a base industry to the whole national economy, for example, in Iceland and in Greenland[28]. The estimated indirect and induced economic impacts of climate change on the fisheries sector of each fishing country were estimated by applying the national fishing output multipliers, M_i, reported in Dyck and Sumaila[29] (Table S6).

$$Economic\ Impact = Total\ Revenue * M_i \tag{13}$$

The GDP of each country in 2010 was obtained from International Monetary Fund (IMF), World Economic Outlook Database (https://www.imf.org/external/pubs/ft/weo/2014/02/weodata/index.aspx). The total revenue is the

current actual landed values obtained from *Sea Around Us* and Fisheries Economics Research Unit price database[17]. Then, the percentage of the economic impact by the fisheries sector to the total GDP of a country was computed to represent the dependency of a country's national economy on fisheries (Table S7).

Human Development Index

In this study, the Human Development Index (HDI) (http://hdr.undp.org/en), which is a composite index measuring average achievement in the 3 basic dimensions of human development (i.e., a long and healthy life, knowledge and a decent standard of living), is used to represent the adaptive capacity of a country to climate change. The HDI of the most recent year available (2013) was obtained from the United Nations Development Programme (http://hdr.undp.org/en/content/human-development-index-hdi-table) (Table S7).

Table S6. National fishing output multipliers by country[29].

Country	Output multiplier	Country	Output multiplier
Albania	1.63	Cayman Is	1.22
Algeria	1.19	Chile	2.44
American Samoa	3.34	China Main	3.34
Angola	3.54	Colombia	3.14
Anguilla	1.21	Comoros	2.95
Antigua Barb	1.22	Congo Dem Rep	3.53
Argentina	2.97	Congo Rep	2.96
Aruba	1.23	Cook Is.	3.34
Australia	3.69	Costa Rica	2.16
Bahamas	1.22	Cote d'Ivoire	1.52
Bahrain	1.02	Croatia	3.27
Bangladesh	2.97	Cuba	1.22
Barbados	1.21	Cyprus	0.61
Belgium	6.22	Denmark	3.72
Belize	3.46	Djibouti	3.00
Benin	1.52	Dominica	1.22
Bermuda	7.34	Dominican Republic	1.21
Br Virgin Is	1.21	East Timor	2.11
Brazil	2.39	Ecuador	3.25
Brunei Darussalam	2.16	Egypt	2.42
Bulgaria	18.34	El Salvador	3.46
Cambodia	1.73	Equatorial Guinea	2.97
Cameroon	2.96	Eritrea	2.95
Canada	3.30	Estonia	3.81
Cape Verde	1.52	Faeroe Is	2.10

Country	Output multiplier	Country	Output multiplier
Falkland Is	2.12	India	1.36
Fiji	3.34	Indonesia	1.66
Finland	1.56	Iran	1.94
Fr Guiana	2.12	Iraq	1.03
Fr Polynesia	3.34	Ireland	2.15
France	4.11	Israel	1.03
Gabon	2.96	Italy	1.75
Gambia	1.52	Jamaica	1.22
Gaza Strip	1.02	Japan	2.75
Georgia	2.04	Jordan	1.06
Germany	3.28	Kenya	2.95
Ghana	1.52	Kiribati	3.34
Gibraltar	0.00	Korea D P Rp (N Korea)	3.04
Greece	3.31	Korea Rep (S Korea)	2.91
Greenland	7.38	Kuwait	1.02
Grenada	1.21	Latvia	4.31
Guadeloupe	1.21	Lebanon	1.02
Guam	3.36	Liberia	1.52
Guatemala	1.87	Libya	1.19
Guinea	1.52	Lithuania	3.79
Guinea Bissau	1.52	Macau	3.05
Guyana	2.12	Madagascar	2.34
Haiti	1.22	Malaysia	2.58
Honduras	3.46	Maldives	2.97
Hong Kong	2.59	Malta	2.54
Iceland	2.49	Marshall Is	3.34

Country	Output multiplier	Country	Output multiplier
Martinique	1.21	Panama	2.56
Mauritania	1.52	Papua New Guinea	3.34
Mauritius	1.62	Peru	2.95
Mayotte	2.95	Philippines	1.19
Mexico	0.61	Poland	4.27
Micronesia	3.34	Portugal	4.78
Monaco	0.00	Puerto Rico	1.21
Montserrat	1.20	Qatar	1.02
Morocco	2.81	Reunion	2.95
Mozambique	1.83	Romania	3.73
Myanmar	0.85	Russian Fed	2.50
N Marianas	3.35	Samoa	3.34
Namibia	4.82	Sao Tome Prn	2.96
Nauru	3.31	Saudi Arabia	1.02
Netherlands Antilles	1.21	Senegal	2.21
Netherlands	2.95	Serbia Montenegro	2.10
New Caledonia	3.34	Seychelles	2.95
New Zealand	2.58	Sierra Leone	1.52
Nicaragua	1.50	Singapore	4.01
Nigeria	0.28	Slovenia	6.23
Niue	3.35	Solomon Is.	3.34
Norfolk I.	0.00	Somalia	2.95
Norway	3.36	South Africa	3.13
Oman	1.02	Spain	3.86
Pakistan	2.16	Sri Lanka	1.01
Palau	3.34	St Helena	1.53

Country	Output multiplier	Country	Output multiplier
St Kitts and Nevis	1.22	US Virgin Is	1.22
St Lucia	1.22	USA	3.10
St Pierre & Miquelon	7.37	Vanuatu	3.34
St Vincent	1.22	Venezuela	1.06
Sudan	2.95	Viet Nam	3.47
Suriname	2.12	Wallis and Futuna	3.33
Sweden	2.66	Yemen	1.02
Syria	1.02		
Taiwan	3.28		
Tanzania	2.72		
Thailand	2.12		
Togo	1.52		
Tokelau	3.35		
Tonga	3.34		
Trinidad Tobago	1.22		
Tunisia	1.46		
Turkey	1.59		
Turks Caicos	1.21		
Tuvalu	3.34		
UK	4.26		
Ukraine	5.56		
United Arab Emirates	1.02		
Uruguay	2.63		
US Virgin Is	1.22		
USA	3.10		
Vanuatu	3.34		

Table S7. Dependency of a country's economy on fisheries – percentage of economic impact by the fisheries sector to the total Gross Domestic Product (GDP) of each fishing country.

Country	% of economic impact to GDP	HDI	HDI Level
Albania	0.02	0.72	High
Algeria	0.13	0.72	High
American Samoa	0.04	0.70	Medium
Angola	0.96	0.53	Low
Anguilla (UK)	1.01	0.77	High
Antigua & Barbuda	0.23	0.77	High
Argentina	0.85	0.81	Very high
Aruba (Netherlands)	0.05	0.79	High
Australia	0.17	0.93	Very high
Azores Isl. (Portugal)	0.44	0.89	Very high
Bahamas	1.66	0.79	High
Bahrain	0.27	0.82	Very high
Bangladesh	0.32	0.56	Medium
Barbados	0.03	0.78	High
Belgium	0.17	0.88	Very high
Belize	3.79	0.73	High
Benin	0.18	0.48	Low
Bermuda (UK)	0.51	0.91	Very high
Bosnia & Herzegovina	0.00	0.73	High
Brazil	0.06	0.74	High
British Virgin Isl. (UK)	0.57	0.84	Very high
Brunei Darussalam	0.01	0.85	Very high
Bulgaria	0.23	0.78	High
Cambodia	0.44	0.58	Medium
Cameroon	0.55	0.50	Low
Canada	0.42	0.90	Very high
Cape Verde	3.06	0.64	Medium
Cayman Isl. (UK)	0.00	0.87	Very high
Chile	3.49	0.82	Very high
China	0.26	0.72	High
Colombia	0.04	0.71	High

Country	% of economic impact to GDP	HDI	HDI Level
Comoros	11.20	0.49	Low
Congo (ex-Zaire)	0.10	0.56	Medium
Congo, R. of	0.70	0.34	Low
Cook Islands	2.57	0.71	High
Costa Rica	0.18	0.76	High
Côte d'Ivoire	0.17	0.45	Low
Croatia	0.22	0.81	Very high
Cuba	0.15	0.81	Very high
Curacao	0.31	0.81	Very high
Denmark	0.85	0.90	Very high
Djibouti	0.00	0.47	Low
Dominica	5.62	0.72	High
Dominican Republic	0.01	0.70	High
Ecuador	0.62	0.71	High
Egypt	0.04	0.68	Medium
El Salvador	0.06	0.66	Medium
Equatorial Guinea	0.40	0.56	Medium
Eritrea	0.10	0.38	Low
Estonia	1.65	0.84	Very high
Faeroe Isl. (Denmark)	25.87	0.86	Very high
Falkland Isl. (UK)	48.59	0.86	Very high
Fiji	1.46	0.72	High
Finland	0.03	0.88	Very high
France	0.09	0.88	Very high
French Guiana	0.02	0.80	Very high
French Polynesia	16.37	0.78	High
Gabon	0.88	0.67	Medium
Gambia	2.96	0.44	Low
Georgia	0.20	0.74	High
Germany	0.03	0.91	Very high
Ghana	1.50	0.57	Medium
Greece	0.66	0.85	Very high
Greenland	51.10	0.79	High
Grenada	0.66	0.74	High

Country	% of economic impact to GDP	HDI	HDI Level
Guadeloupe (France)	0.06	0.83	Very high
Guam (USA)	0.02	0.77	High
Guatemala	0.02	0.63	Medium
Guinea	1.53	0.39	Low
Guinea-Bissau	2.51	0.40	Low
Guyana	3.60	0.64	Medium
Haiti	0.09	0.47	Low
Honduras	0.45	0.62	Medium
Hong Kong	0.13	0.89	Very high
Iceland	22.71	0.89	Very high
India	0.06	0.59	Medium
Indonesia	0.29	0.68	Medium
Iran	0.30	0.75	High
Iraq	0.00	0.64	Medium
Ireland	0.45	0.90	Very high
Israel	0.00	0.89	Very high
Italy	0.15	0.87	Very high
Jamaica	0.03	0.72	High
Japan	0.37	0.89	Very high
Jordan	0.00	0.75	High
Kenya	0.03	0.54	Low
Kiribati	22.51	0.61	Medium
Korea (North)	2.36	0.54	Low
Korea (South)	0.61	0.89	Very high
Kuwait	0.00	0.81	Very high
Latvia	0.69	0.81	Very high
Lebanon	0.00	0.77	High
Liberia	0.91	0.41	Low
Libya	0.11	0.78	High
Lithuania	0.78	0.83	Very high
Madagascar	1.25	0.50	Low
Madeira Isl. (Portugal)	0.27	0.57	Medium
Malaysia	0.82	0.77	High
Maldives	17.97	0.70	Medium

Country	% of economic impact to GDP	HDI	HDI Level
Malta	0.24	0.83	Very high
Marshall Isl.	52.15	0.57	Medium
Martinique (France)	0.11	0.84	Very high
Mauritania	7.01	0.49	Low
Mauritius	0.21	0.77	High
Mayotte (France)	3.21	0.69	Medium
Mexico	0.05	0.76	High
Micronesia	34.89	0.65	Medium
Montenegro	0.03	0.79	High
Montserrat (UK)	0.14	0.64	Medium
Morocco	2.21	0.62	Medium
Mozambique	0.10	0.39	Low
Myanmar	0.10	0.52	Low
Namibia	11.42	0.62	Medium
Nauru	2.03	0.63	Medium
Netherlands	0.24	0.92	Very high
New Caledonia (France)	0.24	0.78	High
New Zealand	1.45	0.91	Very high
Nicaragua	0.40	0.61	Medium
Nigeria	0.02	0.50	Low
Niue (New Zealand)	11.76	0.64	Medium
North Cyprus	0.12	0.85	Very high
North Marianas (USA)	0.03	0.77	High
Norway	1.05	0.94	Very high
Oman	0.28	0.78	High
Pakistan	0.33	0.54	Low
Palau	2.33	0.77	High
Panama	1.72	0.77	High
Papua New Guinea	3.58	0.49	Low
Peru	13.86	0.74	High
Philippines	0.43	0.66	Medium
Poland	0.09	0.83	Very high
Portugal	0.75	0.82	Very high
Puerto Rico (USA)	0.01	0.87	Very high

Country	% of economic impact to GDP	HDI	HDI Level
Qatar	0.01	0.85	Very high
Réunion (France)	0.11	0.82	Very high
Romania	0.03	0.78	High
Russian Federation	0.30	0.78	High
Saint Helena (UK)	6.88	0.65	Medium
Saint Kitts & Nevis	0.12	0.75	High
Saint Lucia	0.20	0.71	High
Saint Vincent & the Grenadines	2.80	0.72	High
Samoa	3.17	0.69	Medium
Sao Tome & Principe	14.93	0.56	Medium
Saudi Arabia	0.04	0.84	Very high
Senegal	13.54	0.49	Low
Seychelles	7.34	0.76	High
Sierra Leone	2.97	0.37	Low
Singapore	0.00	0.90	Very high
Slovenia	0.04	0.87	Very high
Solomon Isl.	16.00	0.49	Low
Somalia	6.57	0.27	Low
South Africa	0.41	0.66	Medium
South Cyprus	0.07	0.85	Very high
Spain	0.53	0.87	Very high
Sri Lanka	0.18	0.75	High
Sudan	0.00	0.47	Low
Suriname	0.67	0.70	High
Sweden	0.07	0.90	Very high
Syrian Arab Republic	0.01	0.66	Medium
Taiwan	0.98	0.88	Very high
Tanzania	0.16	0.49	Low
Thailand	1.06	0.72	High
Timor Leste	0.03	0.62	Medium
Togo	1.19	0.47	Low
Tokelau (New Zealand)	42.48	0.53	Low
Tonga	0.95	0.70	High
Trinidad & Tobago	0.07	0.77	High

Country	% of economic impact to GDP	HDI	HDI Level
Tunisia	0.42	0.72	High
Turkey	0.22	0.76	High
Turks & Caicos Isl. (UK)	1.55	0.75	High
Tuvalu	70.25	0.55	Low
Ukraine	0.45	0.73	High
United Arab Emirates	0.03	0.83	Very high
United Kingdom	0.36	0.89	Very high
Uruguay	0.67	0.79	High
US Virgin Isl.	0.09	0.78	High
USA	0.24	0.91	Very high
Vanuatu	38.29	0.62	Medium
Venezuela	0.12	0.76	High
Viet Nam	0.31	0.64	Medium
Wallis & Futuna Isl. (France)	2.66	0.68	Medium
Yemen	0.52	0.50	Low

References

1 Dunne, J. P. *et al.* GFDL's ESM2 global coupled climate-carbon Earth System Models. Part I: Physical formulation and baseline simulation characteristics. *Journal of Climate* **25**, 6646-6665 (2012).

2 Dufresne, J.-L. *et al.* Climate change projections using the IPSL-CM5 Earth System Model: from CMIP3 to CMIP5. *Climate Dynamics* **40**, 2123-2165 (2013).

3 Giorgetta, M. A. *et al.* Climate and carbon cycle changes from 1850 to 2100 in MPI-ESM simulations for the Coupled Model Intercomparison Project phase 5. *Journal of Advances in Modeling Earth Systems* **5**, 572-597 (2013).

4 Van Vuuren, D. P. *et al.* The representative concentration pathways: an overview. *Climatic Change* **109**, 5-31 (2011).

5 Close C, C. W., Hodgson S, Lam V, Watson R, Pauly D. Distribution ranges of commercial fishes and invertebrates. *Fishes in Databases and Ecosystems. Fisheries Centre Research Reports* **14**, 27–37 (2006).

6 Cheung, W. W., Lam, V. W. & Pauly, D. Modelling present and climate-shifted distribution of marine fishes and invertebrates. (2008).

7 Pauly, D. The relationships between gill surface area and growth performance in fish: a generalization of von Bertalanffy's theory of growth. *Meeresforschung* **28**, 251-282 (1981).

8 Pauly, D. & Kinne, O. *Gasping fish and panting squids: oxygen, temperature and the growth of water-breathing animals*. Vol. 22 (International Ecology Institute, 2010).

9 Pörtner, H.-O. Oxygen-and capacity-limitation of thermal tolerance: a matrix for integrating climate-related stressor effects in marine ecosystems. *The Journal of experimental biology* **213**, 881-893 (2010).

10 Cheung, W. W. *et al.* Projecting global marine biodiversity impacts under climate change scenarios. *Fish Fish* **10**, 235-251 (2009).

11 Cheung, W. W., Watson, R. & Pauly, D. Signature of ocean warming in global fisheries catch. *Nature* **497**, 365-368 (2013).

12 Cheung, W. W., Dunne, J., Sarmiento, J. L. & Pauly, D. Integrating ecophysiology and plankton dynamics into projected maximum fisheries catch potential under climate change in the Northeast Atlantic. *ICES J Mar Sci*, doi:10.1093/icesjms/fsr012 (2011).

13 Zeller, D. *et al.* Still catching attention: Sea Around Us reconstructed global catch data, their spatial expression and public accessibility. *Marine Policy* **70**, 145-152 (2016).

14 Jones, M. C. & Cheung, W. W. Multi-model ensemble projections of climate change effects on global marine biodiversity. *ICES Journal of Marine Science: Journal du Conseil*, fsu172 (2014).

15 Cheung, W. W. L. *et al.* Structural uncertainty in projecting global fisheries catches under climate change. *Ecol. Model.* **325**, 57 - 66 (2016).

16 Sumaila, U. R., Marsden, A. D., Watson, R. & Pauly, D. A global ex-vessel fish price database: construction and applications. *J. Bioecon.* **9**, 39-51 (2007).

17 Swartz, W., Sumaila, R. & Watson, R. Global Ex-vessel Fish Price Database Revisited: A New Approach for Estimating 'Missing' Prices. *Environ. Resour. Econ.*, 1-14, doi:10.1007/s10640-012-9611-1 (2012).

18 Murawski, S. A. & Serchuk, F. M. Mechanized shellfish harvesting and its management: the offshore clam fishery of the eastern United States. *Marine invertebrate fisheries: their assessment and management. Wiley, New York*, 479-506 (1989).

19 Division, D. F. & Fisheries, D. C. f. *Towards sustainable fisheries: economic aspects of the management of living marine resources*. Vol. 1 (OECD, 1997).

20 Asche, F., Bremnes, H. & Wessells, C. R. Product aggregation, market integration, and relationships between prices: an application to world salmon markets. *American Journal of Agricultural Economics* **81**, 568-581 (1999).

21 Hannesson, R. Globalisation of seafood prices: some examples. *ACP-EU Fisheries Research Report* **5**, 15-16 (1999).

22 Pinnegar, J. K., Hutton, T. P. & Placenti, V. What relative seafood prices can tell us about the status of stocks. *Fish and Fisheries* **7**, 219-226 (2006).

23 Alexandratos, N. *World agriculture: towards 2010: an FAO study*. (Food & Agriculture Org., 1995).

24 Sverdrup-Jensen, S. Fish demand and supply projections. *Naga, the ICLARM Quarterly* **20**, 77-78 (1997).

25 Delgado, C. L. & Courbois, C. Changing fish trade and demand patterns in developing countries and their significance for policy research. (International Food Policy Research Institute (IFPRI), 1997).

26 Delgado, C. L. *Fish to 2020: Supply and demand in changing global markets*. Vol. 62 (WorldFish, 2003).

27 Pontecorvo, G., Wilkinson, M., Anderson, R. & Holdowsky, M. Contribution of the ocean sector to the United States economy. *Science* **208**, 1000-1006 (1980).

28 Arnason, R. Climate change and fisheries: assessing the economic impact in Iceland and Greenland. *Natural Resource Modeling* **20**, 163-197 (2007).

29 Dyck, A. J. & Sumaila, U. R. Economic impact of ocean fish populations in the global fishery. *J. Bioecon.* **12**, 227-243 (2010).
